# Studyholism: A New Obsessive-Compulsive Related Disorder? An Analysis of Its Association With Internalizing and Externalizing Features

**DOI:** 10.3389/fpsyg.2021.734116

**Published:** 2022-01-24

**Authors:** Yura Loscalzo, Marco Giannini

**Affiliations:** Department of Health Sciences, School of Psychology, University of Florence, Florence, Italy

**Keywords:** heavy study investment, obsession, OCD, study, study addiction, study engagement, work addiction, workaholism

## Abstract

Studyholism (or obsession toward study) is a new potential obsessive-compulsive (OCD)-related disorder recently introduced in the literature. According to its theorization, there are two types of Studyholic: Engaged and Disengaged Studyholics, which are characterized, respectively, by high and low levels of Study Engagement. This study aims to shed light on the role of internalizing and externalizing features as antecedents and outcomes of Studyholism and Study Engagement. Moreover, it aims to analyze the differences in psychopathology and sensation seeking between students demonstrating Disengaged and Engaged Studyholism. We performed four path analyses, MANOVAs, and Mann–Whitney tests on 1,223 Italian college students (*M*_*age*_ = 22.56 ± 3.53). Among the main findings, Studyholism is associated with psychological and academic impairment, while Study Engagement predicts better mental health and academic functioning; though, the β values are lower for Study Engagement. Moreover, Studyholism is positively predicted by internalizing symptoms and negatively predicted by externalizing variables. Finally, students showing Engaged Studyholism have lower levels of obsessive-compulsive symptoms than those demonstrating Disengaged Studyholism. In conclusion, this study shows the critical importance of implementing preventive interventions aimed at reducing Studyholism levels in college students. Moreover, it provides support to the conceptualization of problematic overstudying as a new potential OCD-related disorder and to the value of distinguishing between Engaged and Disengaged Studyholics for tailored clinical interventions. Finally, it highlights the need to use two different theorizations and operationalizations for problematic overworking and overstudying. However, the literature on problematic overstudying is too scant to reach any firm conclusion. Hence, future studies should deepen the analysis of problematic overstudying, possibly using longitudinal designs, to unveil its internalizing and/or externalizing nature.

## Introduction

Fifty years ago, Oates (1971) introduced the term “Workaholism” to describe people who feel a compulsion to work for a long time and face adverse consequences due to this problem behavior. Since then, many scholars have analyzed Workaholism and its negative outcomes, such as depressive mood, work-family conflict, and poor performance (Sussman, 2012; Clark et al., 2016). Despite the vast literature on the topic, Loscalzo and Giannini (2017a) highlighted the lack of a definition shared by the scientific community; hence, they reviewed the studies on Workaholism and suggested a comprehensive model enclosing all the main features highlighted by different scholars. Therefore, they defined Workaholism as being characterized by both addiction and obsessive-compulsive symptoms, and by either high or low levels of work engagement (hence distinguishing between engaged and disengaged workaholics). In other words, Loscalzo and Giannini (2017a) suggested that Workaholism is made up of both externalizing and internalizing symptoms. Externalizing disorders are characterized by symptoms addressed toward others and low self-control. The feature of internalizing disorders is that symptoms are not shown to others, and there is excessive self-control (Strepparava and Iacchia, 2012). Hence, addiction symptoms might be classified as externalizing, while obsessive-compulsive symptoms might be classified as internalizing. Finally, based on a thorough review of the literature, Loscalzo and Giannini (2017a) listed some potential antecedents and outcomes of Workaholism, distinguishing between individual and situational ones.

Next, in line with Atroszko et al. (2015), Loscalzo and Giannini (2017b) suggested that a problem behavior similar to Workaholism might be evident in the school context too since studying is the main work activity of students. Though, as highlighted in a subsequent paper, Loscalzo and Giannini (2020a) believed that there is a critical difference between work and study. While working is a paid activity, studying (despite a few exceptions) is not rewarded with money. Hence, in their view, it is not appropriate to straightforwardly use the Workaholism construct in the school context; a specific construct for students should be suggested. Therefore, they introduced in the literature a new *potential* clinical condition specifically related to study behavior, namely Studyholism (or obsession toward studying). More specifically, when theorizing this new construct – based on their comprehensive workaholism model (Loscalzo and Giannini, 2017a) – Loscalzo and Giannini (2017b) first defined it as a potential new clinical condition that *might* include both addiction and obsessive symptoms, and either high or low study engagement. Though, based on the psychometric analyses performed on a pool of 68 items covering addiction symptoms, obsessive symptoms, and study engagement (Loscalzo et al., 2018), Loscalzo and Giannini (2017b) next proposed a two-factor definition, not including addiction items. Hence, they defined Studyholism as characterized by obsessive-compulsive symptoms and either high or low Study Engagement. Moreover, they suggested that the clinical form of Studyholism is the one associated with low Study Engagement (or Disengaged Studyholism).

**FIGURE 1:**
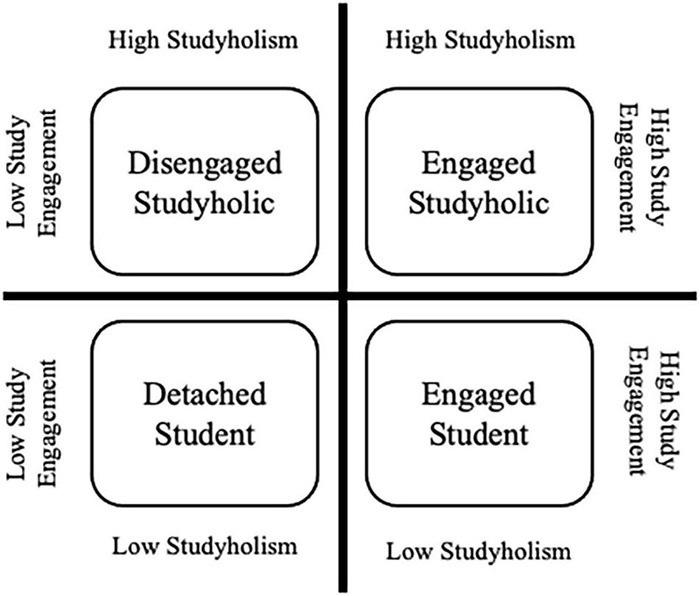
The four types of student accordingly to Loscalzo and Giannini’s (2017b) conceptualization.

Loscalzo and Giannini (2017b) adopted the Heavy Study Investment (HSI) framework. More specifically, referring to the heavy work investment model (Snir and Harpaz, 2012), they defined HSI as a heavy investment of time and effort in studying that might take three different forms (based on the high/low levels of Studyholism and Study Engagement): Disengaged Studyholics (i.e., students with high levels of Studyholism and low levels of Study Engagement), Engaged Studyholics (i.e., students with high Studyholism but also high Study Engagement), and Engaged students (i.e., students with low Studyholism and high Study Engagement). Figure 1 shows graphically the four types of student who arise by crossing the levels of Studyholism and Study Engagement, hence also including Detached students (or students with low levels of both Studyholism and Study Engagement). In Loscalzo and Giannini’s (2017b) perspective, it is vital to adopt the HSI framework for two main reasons: (i) To avoid over-pathologizing a common behavior such as studying (in line with Billieux et al., 2015); (ii) To detect potential differences between different types of Studyholics with regard to the same antecedents/outcomes, with critical implications for preventive and clinical interventions.

Loscalzo and Giannini (2017b), in their first theoretical paper, specified that they chose to name this new potential clinical condition as “Studyholism” since they aimed to maintain continuity with the construct of Workaholism, as they hypothesized that Studyholism could be an antecedent of it. Also, using a term that does not include the word “addiction” is vital for Loscalzo and Giannini (2017b), as it helps avoid a reduction of the construct to the addiction component, giving the possibility to consider the copresence of a positive dimension (namely, study engagement). Loscalzo and Giannini (2017b) introduced Studyholism as a construct different from Study Addiction (Atroszko et al., 2015), even if they are both related to problematic overstudying. “Problematic overstudying” is a general term coined by Loscalzo and Giannini (2018a) to refer to the analysis of this problematic behavior regardless of its theorization. Instead, Studyholism (Loscalzo and Giannini, 2017b) and Study Addiction (Atroszko et al., 2015) refer to problematic overstudying in the context of a specific model: the obsessive-compulsive (OCD)-related disorder and the behavioral addiction model, respectively. Atroszko et al. (2015) defined Study Addiction as a behavioral addiction characterized by seven core components of substance addictions (i.e., salience, tolerance, mood modification, relapse, withdrawal, conflict, and problems). Concerning terminology, it should also be considered that when referring to Studyholism (without specifying if it is Engaged or Disengaged Studyholism), we consider only study-related obsessive symptoms, without taking into account the levels of Study Engagement.

After the first publication by Loscalzo and Giannini (2017b), the definition of problematic overstudying as a condition *more similar to* an obsession—or Studyholism (e.g., Loscalzo and Giannini, 2017b,2018a,2018b)—than to addiction (i.e., Study Addiction; Atroszko et al., 2015), or as an OCD-related disorder, has been substantiated by a thorough comparison of DSM-5 (American Psychiatric Association [APA], 2013) diagnostic criteria for OCD, substance-use disorder, and obsessive-compulsive personality disorder (Loscalzo and Giannini, 2018a). Moreover, worry, an internalizing feature contributing to OCD (Comer et al., 2004), proved to be a strong predictor of Studyholism both in college (Loscalzo and Giannini, 2019a) and adolescent (Loscalzo, 2021) students. Though, the authors concluded these papers by underlining that the literature concerning problematic overstudying is too scant to reach any firm conclusion and that more studies are needed to uncover its real internalizing and/or externalizing nature.

In line with this, since Loscalzo and Giannini believed that unveiling the real nature of a new potential clinical condition requires avoiding a confirmatory approach [refer to Kardefelt-Winther (2015) concerning the need to avoid an *a priori* assumption of addiction when analyzing new potential behavioral addictions], they reviewed some elements of their preliminary definition (Loscalzo and Giannini, 2017b) based on their empirical findings. In fact, the results of the outcomes associated with Engaged and Disengaged Studyholism showed that students demonstrating Disengaged Studyholism are not the most impaired type of students in all the functional areas (Loscalzo and Giannini, 2019a; Loscalzo, 2021). Hence, Loscalzo and Giannini (2019a) suggested conceptualizing both Disengaged and Engaged Studyholics as clinical types of Studyholism and using the following two specifiers: (i) Level of study engagement (high, average, or low); (ii) Area of functional impairment (academic, social, or both).

In conclusion, Loscalzo and Giannini (2020a) suggested these tentative DSM-like Studyholism criteria: Studyholism is characterized by persistent and recurrent problematic studying behaviors that produce clinically significant impairment/distress and, more specifically, by study-related obsessions and/or study-related compulsions during the last 6 months. Also, Loscalzo and Giannini included the usual DSM-5 (American Psychiatric Association [APA], 2013) exclusion criteria (i.e., physiological effects of a substance or medical condition and other mental disorders). Finally, they foresaw two specifiers: (i) Study engagement level (high, average, or low); (ii) Main area of impairment (academic, social, or both: academic and social). However, Loscalzo and Giannini (2020a) stated that future quantitative and qualitative studies should analyze if these tentative criteria are suitable for an accurate definition of Studyholism and delete or add criteria, if needed. Therefore, a comprehensive analysis of the relationship between Studyholism, Study Engagement, psychopathology, and sensation seeking constitutes a critical step in providing evidence concerning the internalizing and/or externalizing nature of problematic overstudying.

Even if Studyholism is not recognized as a clinical disorder, it is critical to analyze it further since previous studies showed that it is widespread in Italian youths, preadolescents, and adolescents (Loscalzo, 2019; Loscalzo et al., 2021) and that it is associated with negative outcomes in the psychological, physical, academic, and social areas (Loscalzo and Giannini, 2019a; Loscalzo, 2021). Moreover, since 2000, mental health of college students received increasing interest, as many scholars were captivated by the high prevalence of psychopathology in this population. More specifically, previous studies highlighted that mental disorders and high distress are common among university students, even if the onset generally occurs before the beginning of college (e.g., Megivern et al., 2003; Rosenthal and Wilson, 2008; Storrie et al., 2010; Auerbach et al., 2016). Depression, anxiety, suicidal ideation, and self-injury are widespread clinical diagnoses in college students (e.g., Blanco et al., 2008; Gallagher, 2008; Drum et al., 2009; Eisenberg et al., 2013; Ibrahim et al., 2013). Hence, professionals should be aware of the high prevalence of mental disorders in college students, including high levels of Studyholism, as this population might efficiently receive preventive and treatment interventions. In line with this, Regehr et al. (2013) urged universities to offer students interventions aimed at reducing stress (such as cognitive, behavioral, and mindfulness programs), also considering the negative outcomes associated with mental issues, including lower Grade Point Average (GPA) and lower rates of graduations, compared to peers not suffering from mental health disorders (e.g., Keyes et al., 2012; Salzer, 2012).

Though, the literature specifically related to problematic overstudying is scant, and the studies about its relationships with clinical diagnoses are almost absent. To the best of the authors’ knowledge, there is only one study by Lawendowski et al. (2020). This study analyzed the relationships between study addiction and social anxiety on a sample of 132 students of Polish music academies. Through a regression analysis, they concluded that social anxiety is a predictor of study addiction (β = 0.24, *p* = 0.017). Hence, this study has the merit of shedding light on the role of an internalizing disorder as a contributing factor of problematic overstudying. However, the sample is small and representative of a particular type of student (musicians). Moreover, the theoretical framework is that of behavioral addictions.

Given the lack of literature concerning problematic overstudying, it might be helpful to ground on the literature about problematic overworking. Loscalzo and Giannini (2020b) recently analyzed the role of psychopathology (evaluated through the Symptom Check List-90-R; Derogatis, 1994) as an antecedent and outcome of Workaholism and Work Engagement, as well as the role of sensation seeking as an antecedent. Zuckerman (1994) defined sensation seeking as a personality trait whose main features are as follows: (i) The seeking of experiences and situations which are varied, novel, complex, and intense; (ii) The willingness to face the issues which might be associated with these experiences, such as physical, social, financial, and legal issues. This personality trait characterizes people who use (and abuse) drugs, alcohol, and marijuana (e.g., Linden-Carmichael et al., 2016; Meil et al., 2016; Rogers et al., 2018). Therefore, aiming to shed light on the internalizing and/or externalizing nature of Workaholism, Loscalzo and Giannini (2020b) included sensation seeking among the externalizing variables that could predict Workaholism. Their results showed that Workaholism predicts higher internalizing and externalizing symptoms (while Work Engagement is a negative predictor of all the SCL-90-R scales). Moreover, the predictors of Work Engagement are depression and boredom susceptibility (negative predictors) and somatization (positive predictor). Workaholism, instead, is positively predicted only by psychoticism. Based on these results, Loscalzo and Giannini (2020b) suggested that Workaholism might be defined as a declination at work of a personality disorder (like the Schizoid or Obsessive-Compulsive) and the importance to analyze other explanations besides addiction, also taking into account that sensation seeking, a feature of externalizing disorders, including substance addictions (e.g., Linden-Carmichael et al., 2016; Meil et al., 2016; Rogers et al., 2018), does not predict Workaholism. About Work Engagement, the authors suggested that it might be a coping strategy with somatization symptoms.

Besides this comprehensive study, there are a few others that addressed some psychological symptoms (mostly somatization, depression, anxiety, or generic mental health) and usually did not address psychopathology as an antecedent of Workaholism (e.g., Bartcazk and Ogińska-Bulik, 2012; Nie and Sun, 2016; Andreassen et al., 2018). Moreover, only a few studies included Work Engagement in the analyses (e.g., Andreassen et al., 2007; Haar and Roche, 2013). In sum, as previously reviewed by Loscalzo and Giannini (2020b), (i) There are a few studies supporting the association between Workaholism and somatization, anxiety, depression, attention deficit and hyperactivity disorder (ADHD), and OCD symptoms; (ii) There is preliminary evidence for ADHD and anxiety as predictors of Workaholism, in contrast with depression and OCD symptoms; (iii) Work Engagement is associated with lower somatization, anxiety, and depression, even if Shimazu et al. (2018) found that higher levels of Work Engagement may harm mental health in the short-term (though this negative effect disappears, and it becomes positive, in the long-term).

Given the scant literature addressing the internalizing and/or externalizing nature of problematic overstudying and overworking, and the directionality of the relationships between various psychopathology symptoms and problematic overstudying, the present study is of critical importance. First, Study 1 will shed light on the role of Studyholism and Study Engagement in predicting psychopathology. Second, Study 2 will give information about the internalizing and/or externalizing nature of problematic overstudying. Third, employing the same scales used by Loscalzo and Giannini (2020b) with workers, it will allow for comparing the results on workers and students. Hence, it will give further insight into the assertion of Loscalzo and Giannini (2017b,2019b) that Workaholism and Studyholism are two different constructs that, despite having some similarities, have their features, and hence must be conceptualized through different theories [instead of using a straightforward application of the theoretical and empirical framework of work addiction, as done by Atroszko et al. (2015)].

In sum, this study focuses on the role of internalizing and externalizing psychopathology as both an antecedent and an outcome of Studyholism and Study Engagement; moreover, it aims to analyze the role of sensation seeking as an antecedent of Studyholism and Study Engagement. Finally, in line with Loscalzo and Giannini (2020b), we included a performance variable among the outcomes: the number of exams given (regardless of their outcome). Previous studies showed that Studyholism is not a predictor of GPA (or has a low value of negative correlation), in contrast with Study Engagement (Loscalzo and Giannini, 2019a,2020a,2020c; Loscalzo, 2021). Hence, it is interesting to analyze how the two types of HSI predict a different academic indicator. Finally, we explore differences in psychopathology and sensation seeking between students with high/low levels of Studyholism/Study Engagement and between students showing Disengaged and Engaged Studyholism. Since the literature concerning the variables under analysis is very meager, we did not posit hypotheses regarding each specific internalizing and externalizing variable included in the model. Though, based on the comprehensive model of Loscalzo and Giannini (2017b), which suggests psychopathology as both an antecedent and an outcome of Studyholism, and based on the few previous studies about Studyholism and Study Engagement (Loscalzo and Giannini, 2019a; Loscalzo, 2021), we expect that Studyholism is a positive predictor of internalizing and externalizing psychopathology, while Study Engagement predicts lower levels of psychological symptoms. About sensation seeking, in line with Loscalzo and Giannini (2020b) and with the previous studies supporting the definition of Studyholism as an OCD-related disorder (Loscalzo and Giannini, 2019a; Loscalzo, 2021), we expect that it will not be a statistically significant predictor of Studyholism.

## Materials and Methods

### Participants

We got the participation of 1,223 Italian college students aged between 18 and 50 years (*M* = 22.56 ± 3.53). Most of the participants were women (70.4%), lived in Tuscany (65.2%), and were not involved in a job besides studying (77.3%). Considering the length of the instruments selected for comprehensively evaluating psychopathology and sensation seeking, we designed two studies. For Study 1, we administered only the psychopathology scale (*n* = 506). Next, for Study 2, we administered both the psychopathology scale and the scale assessing sensation seeking (*n* = 717). For both the studies, we also administered two scales aimed at evaluating Studyholism and Study Engagement.

The first sample of participants comprises 506 students (70.6% women) aged between 18 and 49 (*M* = 21.24 ± 3.08). All the students lived in Tuscany and attended a course at the University of Florence. Regarding their professional status, most participants did not work besides studying (84%). Concerning their civil status, most of them were engaged (49.8%) or single (45.5%). There were also some cohabitants (2.4%) and just a few married (0.6%) (there were some missing cases). Concerning the study area, most participants were students of Psychology (37.7%) or students of Health Professions (19.8%). However, other areas of study are represented, such as Social and Political Sciences (17.4%) and Engineering (12.5%). Finally, with regard to the year of study, most of the sample was made up of first-year (48.8%) and third-year (42.9%) students. We used this first sample to analyze psychopathology as an outcome of Studyholism and Study Engagement.

The second sample of participants comprises 717 students (70.2% women) aged between 18 and 50 (*M* = 23.50 ± 3.52). The 40.6% of participants lived in Tuscany, while the others lived across other Italian regions. Regarding their professional status, most participants did not work besides studying (72.5%). Finally, concerning their civil status, singles (47.8%) and engaged (43.7%) are the categories most represented, followed by cohabitant (6.6%), married (1.7%), and a few separated (0.1%) or divorced (0.1%). Concerning the area and year of study, this sample is more heterogeneous compared to the first sample. Among the areas of study most represented, there were Engineering (18.1%), Medical studies (13.2%), Literature and Languages (11.4%), Economy (8.8%), Psychology (8.1%), Law (6.6%), and Educational studies (4.2%). Though, there were also students from other courses, such as Health Professions (3.3%), Social and Political Sciences (3.1%), History and Philosophy (2.1%), Maths, Physics and Natural Sciences (2.1%), Chemical studies (2.0%), and Biology (1.3%). Finally, concerning the year of study, the following were the percentages, respectively, from the first to the sixth (for medical students only) years: 11.3, 19.7, 25.7, 15.2, 24.4, and 3.8%. We used this second sample for the analysis concerning psychopathology and sensation seeking as antecedents of Studyholism and Study Engagement.

### Materials

#### Studyholism Inventory (SI-10)

The SI-10 is a brief screening instrument created by Loscalzo et al. (2018) from a pool of 68 items. It comprises two scales, each one comprehending four items plus one filler: Studyholism and Study Engagement. The response format is a 5-point Likert scale ranging between 1 (*Strongly Disagree*) and 5 (*Strongly Agree*). The SI-10 also has a head-sheet with some questions about study habits (e.g., GPA, time spent studying daily and weekly generally and before exams). Currently, the SI-10 is available in Italian, English, Polish, Spanish, and Croatian translations. In this study, we administered the Italian version, which proved to have good psychometric properties (Loscalzo et al., 2018; Loscalzo and Giannini, 2020c).

#### Studyholism Inventory – Extended Version (SI-15)

Loscalzo and Giannini (2020a) created, from a pool of 45 items, an extended version of the SI-10 Studyholism scale, aiming to deepen the measurement of Studyholism. In fact, in its 15-item and 3-factor final version, the SI-15 evaluates Studyholism through three scales: Obsessions (also addressed by the SI-10), Compulsions, and Social Impairment. The Obsessions scale is made up of the four SI-10 Studyholism scale items plus an additional item. The response format is a 5-point Likert scale ranging from 1 (*Strongly Disagree*) to 5 (*Strongly Agree*). The SI-15 is currently available in Italian, English, Polish, and Spanish versions. We administered the Italian version for this study, which has good psychometric properties (Loscalzo and Giannini, 2020a).

#### Symptom Check List-90-R (SCL-90-R)

The SCL-90-R (Derogatis, 1994) is a 90-item self-report instrument that allows for evaluating both internalizing and externalizing symptoms through nine scales: Somatization (the distress arising from bodily perceptions), obsessive-compulsive (the typical obsessive-compulsive symptoms), interpersonal sensitivity (feeling inadequate and inferior to others), depression (including the lack of motivation), anxiety (evaluating both anxiety symptoms and tension), hostility (negative affect, irritability, and aggressiveness), phobic anxiety (persistent fears of specific situations), paranoid ideation (hostility, suspiciousness, projection, and fear of loss of autonomy), and psychoticism (symptoms ranging from mild interpersonal alienation to psychosis). The Italian version foresees a 5-point Likert scale ranging from 1 (*not at all*) to 5 (*extremely*).

#### Sensation Seeking Scale Form V (SSS-V)

The SSS-V (Zuckerman et al., 1978; Zuckerman, 1994) is a 40-item self-report scale for the evaluation of sensation seeking. It is made up of four scales: thrill and adventure-seeking (the desire to engage in dangerous sport or physical activities), experience seeking (looking for new experiences involving mind and senses and having a non-conformist lifestyle), disinhibition (the interest in disinhibited social and sexual activities), and boredom susceptibility (the repulsion for routine and repetitive activities). Each scale comprises 10 items, and each item presents two sentences (A and B); hence, the participants answer by choosing the sentence that applies the best to them. The scoring foresees to give a “1” if the sentence represents sensation seeking and a “0” to the other sentence.

There is not an Italian validation of the SSS-V on an adult population; though, it has been previously used by Tonetti et al. (2010) for a study on Italian youths and by Loscalzo and Giannini (2020b) for their Italian study on sensation seeking as an antecedent of Workaholism and Work Engagement. Hence, we decided to use the SSS-V since it allows us to evaluate sensation seeking in its four dimensions and compare the current study results with the one conducted by Loscalzo and Giannini (2020b) on workers.

### Procedure

First, we asked the approval from the Ethical Committee of the University of Florence. Then, the participants of Study 1 were requested to fill out the paper-and-pencil questionnaire, including the SI-10, the SI-15, and the SCL-90-R, as well as the first page with demographic variables (e.g., gender and age). The health professionals and psychology students filled out the questionnaire during a class. They were free to decline participation in the research, and no credit was given for participation. The other students involved in Study 1 were contacted at their universities, in common spaces, such as libraries and University rooms outside classes. Each participant signed the Informed Consent form before filling out the questionnaire.

For Study 2, we created an online questionnaire containing the SI-10, the SI-15, the SCL-90-R, and SSS-V (besides demographic data). We recruited participants through the spread of the invitation to the research in social networks (the questionnaire itself has not been spread in social networks), aiming to reach participants outside Tuscany and across different areas of study. Since the questionnaire was filled online, we wrote all the information required by the Informed Consent on the first page of the questionnaire, and we asked the participants to check the box stating that by filling the questionnaire on the following pages, they agreed to take part in the research.

All the data have been gathered before the COVID-19 outbreak.

### Data Analysis

We performed the analyses using SPSS 27 and AMOS 22.

First, we analyzed the zero-order correlations of the variables included in the models on the total sample (*n* = 1223). Then, we conducted four Structural Equation Models (SEMs), and more specifically, path analyses (Maximum Likelihood estimate method). For Study 1 (*n* = 506): (i) We performed a path analysis model with the nine psychopathology scales and the number of exams given as outcomes of Studyholism and Study Engagement; (ii) We performed a second path analysis in which the antecedents of psychopathology and the number of exams given were the three SI-15 subscales, namely obsessions, compulsions, and social impairment. Then, for Study 2 (*n* = 717), (iii) We performed a path analysis model with psychopathology and sensation seeking as predictors of Studyholism and Study Engagement; (iv) We performed a last path analysis model with psychopathology and sensation seeking as predictors of the three SI-15 subscales.

To evaluate the fit of the models, we used the following indices and cut-off values: χ^2^/df ratio, which indicates a good fit if its value is less than 3 (Byrne, 2001); goodness of fit index (GFI) and comparative fit index (CFI), whose cut-offs were as follows: less than 0.90 lack of fit, 0.90–0.95 good fit, greater than 0.95 excellent fit (Hu and Bentler, 1999); and Root Mean Square Error of Approximation (RMSEA), where a value below 0.05 indicates excellent fit, while values between 0.05 and 0.08 indicate an acceptable fit (Reeve et al., 2007).

Then, we analyzed differences between high and low Studyholism/Study Engagement in the psychopathology and sensation seeking scales through four MANOVAs. Finally, we analyzed differences between students demonstrating Disengaged and Engaged Studyholism in the psychopathology and sensation seeking scales through two Mann–Whitney tests (with Yates’ continuity correction). The two types of Studyholics (and the high/low levels of Studyholism/Study Engagement) have been created referring to the SI-10 cut-off values for Italian College students (Loscalzo and Giannini, 2020c).

## Results

### Correlations Among the Study Variables

First, we analyzed the zero-order correlations among all the variables included in the path analysis models (Table 1 shows the results of these analyses).

**TABLE 1:** Zero-order correlations among study variables (*n* = 1223 for SCL-90-R scale; *n* = 717 for SSS-V scales).

	1	2	3	4	5	6	7	8	9	10	11	12	13	14	15	16	17	18	19	20	21
1. SH	–																				
2. SE	0.14***	–																			
3. Obs	0.80***	0.15***	–																		
4. Comp	0.31***	0.43***	0.43***	–																	
5. Soc.Imp	0.34***	0.36***	0.46***	0.68***	–																
6. SI-15 Tot	0.61***	0.37***	0.79***	0.84***	0.85***	–															
7. SOM	0.38***	0.003	0.43***	0.19***	0.24***	0.36***	–														
8. O-C	0.49***	−0.13***	0.51***	0.16***	0.19***	0.36***	0.59***	–													
9. I-S	0.39***	−0.07*	0.42***	0.16***	0.24***	0.34***	0.51***	0.70***	–												
10. DEP	0.52***	−0.09***	0.55***	0.20***	0.23***	0.41***	0.64***	0.83***	0.77***	–											
11. ANX	0.49***	0.01	0.54***	0.23***	0.28***	0.43***	0.73***	0.74***	0.68***	0.82***	–										
12. HOS	0.34***	−0.10***	0.32***	0.07*	0.15***	0.23***	0.52***	0.56***	0.56***	0.60***	0.59***	–									
13. PHOB	0.26***	–0.05	0.28***	0.12***	0.19***	0.24***	0.53***	0.53***	0.60***	0.58***	0.63***	0.40***	–								
14. PAR	0.29***	–0.06	0.31***	0.13***	0.17***	0.25***	0.48***	0.60***	0.74***	0.65***	0.60***	0.60***	0.48***	–							
15. PSY	0.31***	−0.11***	0.33***	0.13***	0.17***	0.26***	0.54***	0.69***	0.75***	0.76***	0.71***	0.56***	0.58***	0.73***	–						
16. BS	−0.12***	−0.18***	−0.09*	−0.09*	−0.10**	−0.11**	–0.04	0.05	0.04	0.06	0.01	0.13***	–0.02	0.09*	0.12***	–					
17. DIS	−0.12***	−0.22***	−0.15***	−0.19***	−0.22***	−0.22***	–0.05	0.002	–0.03	–0.02	–0.03	0.09*	−0.10**	0.03	0.06	0.36***	–				
18. ES	−0.14***	−0.13***	−0.15***	−0.10**	−0.11**	−0.15***	–0.07	–0.06	–0.05	–0.07	−0.09*	–0.01	−0.09*	–0.05	0.001	0.21***	0.45***	–			
19. TAS	−0.12**	−0.11**	−0.11**	−0.11**	–0.06	−0.12**	–0.07	–0.07	−0.07*	–0.06	−0.12**	0.01	−0.14***	–0.04	–0.03	0.12***	0.28***	0.37***	–		
20. SSS	−0.18***	−0.23***	−0.18***	−0.18***	−0.17***	−0.22***	−0.08*	–0.03	–0.05	–0.04	−0.09*	0.07	−0.13***	0.01	0.05	0.55***	0.76***	0.71***	0.71***	–	
21. N.Ex	–0.01	0.19***	0.03	0.12**	0.06	0.08	–0.02	−0.14**	–0.02	–0.04	–0.03	−0.12*	–0.08	−0.10*	−0.11*	–0.03	–0.06	–0.04	0.07	–0.01	–

SH, Studyholism (Studyholism Inventory); SE, Study Engagement (Studyholism Inventory); Obs, Obsessions (Studyholism Inventory – Extended Version); Comp, Compulsions (Studyholism Inventory – Extended Version); Soc.Imp, Social Impairment (Studyholism Inventory – Extended Version); SI-15 Tot, Studyholism Inventory – Extended Version, total score; SOM, Somatization; O-C, obsessive-compulsive; I-S, Interpersonal Sensitivity; DEP, Depression; ANX, Anxiety; HOS, Hostility; PHOB, Phobic Anxiety; PAR, Paranoid Ideation; PSY, Psychoticism. All the psychopathology scales are from the Symptoms Checklist 90 Revised (SCL-90-R); BS, Boredom Susceptibility; DIS, Disinhibition; ES, Experience Seeking; TAS, Thrill and Adventure Seeking; SSS, Sensation Seeking total score; All the sensation seeking scales are from the Sensation Seeking Scale Form V (SSS-V); N.Ex, Number of exams given (regardless of the outcome); ***p ≤ 0.001; **p ≤ 0.01; *p ≤ 0.05.

We found that the SI-10 Studyholism scale and the SI-15 scales (obsessions, compulsions, and social impairment) have positive values of correlation with internalizing and externalizing symptoms, though the SI-15 obsessions scale has higher values of correlation compared to the other SI-15 scales. Instead, the SI-10 Study Engagement scale has just a few statistically significant (negative) correlations with the SCL-90-R scales, and the values are generally low. About sensation seeking, both Studyholism scales and Study Engagement correlate negatively with the SSS-V subscales and total score (even if the *r* values are low). There is just a negative correlation that is not statistically significant, even if negative. Finally, the number of exams given does not correlate with the Studyholism scales (except for a low correlation with the SI-15 Compulsion scale), while it positively correlates with Study Engagement.

### Path Analysis Models—Internalizing and Externalizing Symptoms as Outcomes of Heavy Study Investment

**FIGURE 2:**
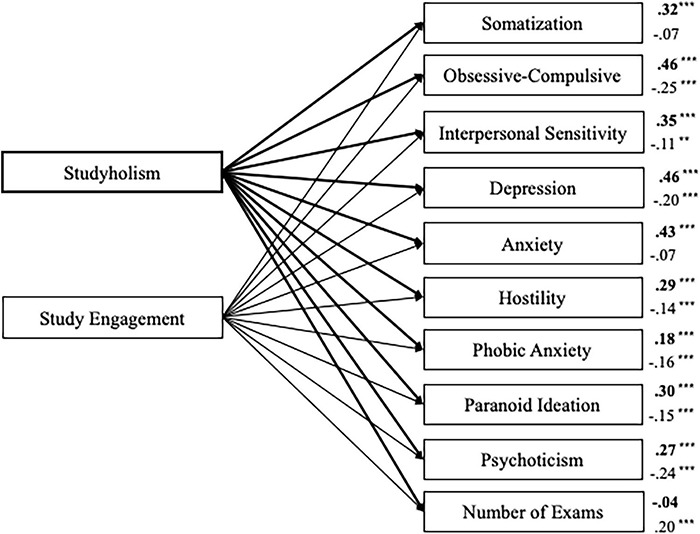
Structural model with standardized path estimates for psychopathology and number of exams taken as outcomes of Studyholism and Study Engagement (*n* = 506). Bold path estimates and lines = Studyholism; Plain path estimates and lines = Study Engagement; ***p < 0.001; **p = 0.012.

First, we tested a model in which the SCL-90-R scales and the number of exams given (regardless of the result) were the outcomes of both Studyholism and Study Engagement (as evaluated through the SI-10 scales; *n* = 506). The model showed an excellent fit to the data: χ^2^/df = 2.32, *p* = 0.013; GFI = 0.99; CFI = 1.00; RMSEA = 0.05, 90% CI = (0.02–0.08). Figure 2 depicts the structural model with standardized path estimates.

In sum, Studyholism is a positive predictor of all the internalizing and externalizing scales, while Study Engagement is a negative predictor of these symptoms (except for somatization and anxiety), even if its β values are lower compared to Studyholism. The number of exams given, instead, is positively predicted only by Study Engagement. The highest β values belong to Studyholism on obsessive-compulsive, depression, and anxiety. The psychopathology scales whose variance is explained the most by Studyholism and Study Engagement include obsessive-compulsive (23.8% of the variance), depression (22.3% of the variance), and anxiety (18.4%). The scale whose variance is explained the least is phobic anxiety (4.8%). The variance explained for the other scales are as follows: interpersonal sensitivity, 12%; psychoticism, 11%; somatization, 9.9%; paranoid ideation, 9.8%; and hostility, 9.0%. The variance explained for the number of exams given is very low (3.8%).

Next, on the same sample (*n* = 506), we tested a second model in which the SCL-90-R scales and the number of exams given are the outcomes of the three SI-15 Studyholism scales.

**FIGURE 3:**
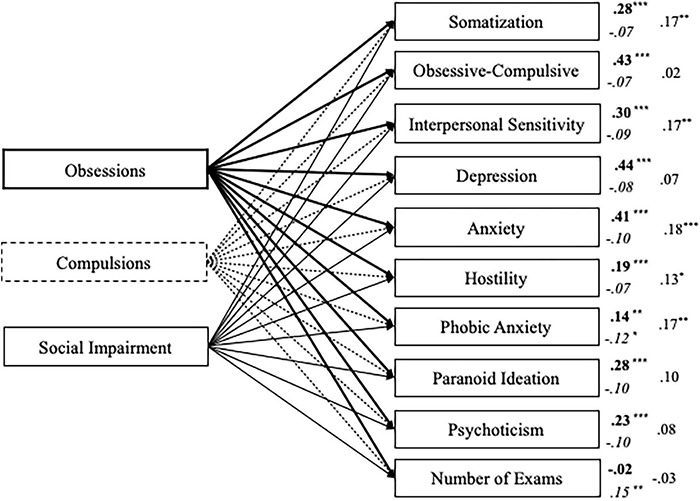
Structural model with standardized path estimates for psychopathology and number of exams taken as outcomes of Obsessions, Compulsions, and Social Impairment (*n* = 506). Bold path estimates and lines = Obsessions; Italics path estimates and dashed lines = Compulsions; Plain path estimates and lines = Social Impairment; ***p < 0.001; **p ≤ 0.01, *p < 0.05.

The model showed a good fit to the data: χ^2^/df = 3.26, *p* = 0.001; GFI = 0.99; CFI = 1.00; RMSEA = 0.07, 90% CI = (0.04–0.10). Figure 3 depicts the structural model with standardized path estimates.

In line with the previous model, the obsessions scale (whose items comprehend the four items of the SI-10 Studyholism scale) is a positive predictor of all the internalizing and externalizing scales, with obsessive-compulsive, depression, and anxiety scales as the ones who are predicted through the highest β values. Also, it does not predict the number of exams taken. About the other SI-15 scales, compulsions show just a few statistically significant paths: phobic anxiety (negative path) and the number of exams (positive path). Finally, social impairment positively predicts five (up to nine) internalizing and externalizing scales, though the β values are low. The psychopathology scales whose variance is explained the most by the SI-15 scales are, also in this model, the following: anxiety (22.3%), depression (19.8%), and obsessive-compulsive (17.3%). Next, there are interpersonal sensitivity (13.5%) and somatization (12.6%). Psychoticism (5.8%), paranoid ideation (9.1%), hostility (5.7%), phobic anxiety (4.7%), and especially, exams given (1.5%) are explained at a lower extent.

### Sensation Seeking and Internalizing and Externalizing Disorders as Antecedents of Heavy Study Investment

**FIGURE 4:**
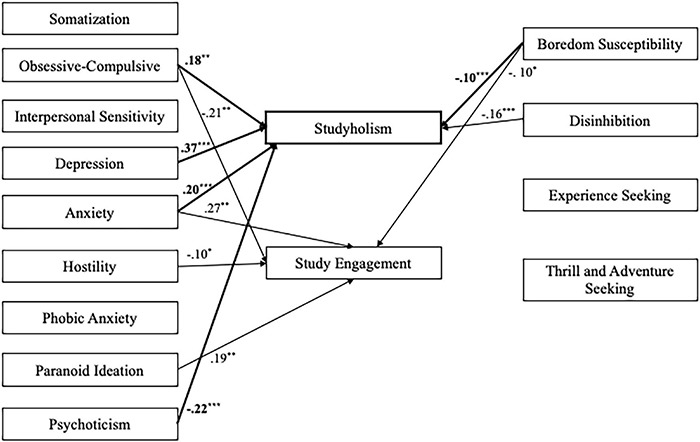
Structural model with standardized path estimates for psychopathology and sensation seeking as antecedents of Studyholism and Study Engagement (*n* = 717). Bold path estimates and lines = Studyholism; Plain path estimates and lines = Study Engagement; The paths that are not statistically significant are not reported; ***p ≤ 0.001; **p < 0.01; *p < 0.05.

Then, on the second sample (*n* = 717), we tested a path analysis model with psychopathology and sensation seeking as predictors of Studyholism and Study Engagement (evaluated through the SI-10 scales). Figure 4 depicts the structural model with the statistically significant standardized path estimates. The model showed an excellent fit to the data: χ^2^/df = 2.60, *p* < 0.001; GFI = 0.98; CFI = 0.99; RMSEA = 0.05, 90% CI = (0.04–0.06). However, the predictors explain a good percent of variance just for Studyholism (39%). For Study Engagement, the explained variance is 12%. Moreover, not all the paths are statistically significant: Studyholism is positively predicted by obsessive-compulsive, depression, and anxiety scales, while psychoticism and boredom susceptibility negatively predict it. Study Engagement is positively predicted by anxiety and paranoid ideation, while it is negatively predicted by obsessive-compulsive, hostility, boredom susceptibility, and disinhibition.

**FIGURE 5:**
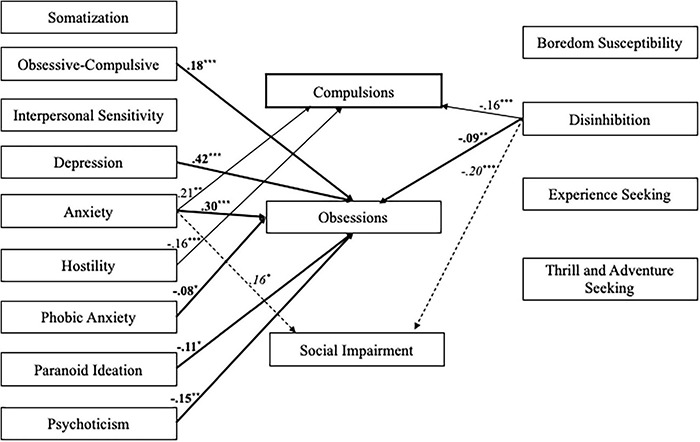
Structural model with standardized path estimates for psychopathology and sensation seeking as outcomes of Obsessions, Compulsions, and Social Impairment (*n* = 717). Bold path estimates and lines = Obsessions; Plain path estimates and lines = Compulsions; Italics path estimates and dashed lines = Social Impairment. The paths that are not statistically significant are not reported ***p ≤ 0.001; **p < 0.01; *p < 0.05.

Finally, we tested a fourth model (*n* = 717) with psychopathology and sensation seeking as predictors of SI-15 Studyholism scales. Figure 5 depicts the structural model with the statistically significant standardized path estimates. The model showed an excellent fit to the data: χ^2^/df = 2.60, *p* < 0.001; GFI = 0.98; CFI = 0.99; RMSEA = 0.05, 90% CI = (0.04–0.06), Though, the predictors explain a good percent of variance just for obsessions (45.8%). For compulsions and social impairment, the variances explained are, respectively, 10.6 and 12.2%. Moreover, just a few paths are statistically significant: Obsessions is positively predicted by obsessive-compulsive, depression, and anxiety scales, while it is negatively predicted by phobic anxiety, paranoid ideation, psychoticism, and disinhibition. About compulsions, they are positively predicted only by anxiety, and they are negatively predicted by hostility and disinhibition. Finally, Social Impairment is positively predicted by anxiety and negatively predicted by disinhibition.

### Differences in Psychopathology and Sensation Seeking Among Students With Different Levels of Studyholism and Study Engagement

We performed two MANOVAs on the total sample (*n* = 1223) to analyze if there are differences in psychopathology between students characterized by high (*n* = 144, 11.80%) and low (*n* = 217, 17.70%) levels of Studyholism, as well as by high (*n* = 118, 9.60%) and low (*n* = 229, 18.70%) levels of Study Engagement.

Concerning Studyholism, the multivariate test highlighted a statistically significant effect on psychopathology: *F*(9,351) = 58.29, *p* < 0.001, η^2^ = 0.60. More specifically, follow-up ANOVAs showed statistically significant differences in all the SCL-90-R scales: students demonstrating high levels of Studyholism have higher scores on all the psychopathology scales compared to students demonstrating low levels of Studyholism. About Study Engagement, we found again a multivariate statistically significant effect: *F*(9,337) = 6.79, *p* < 0.001, η^2^ = 0.15. However, follow-up ANOVAs showed that there is a statistically significant difference just for a few SCL-90-R scales; more specifically, students showing high Study Engagement have lower levels of obsessive-compulsive symptoms, interpersonal sensitivity, depression, hostility, and psychoticism. Table 2 shows the results of follow-up ANOVAs analyses.

**TABLE 2:** Follow-up ANOVAs.

SCL-90-R		Level	*n*	*M (SD)*	*F* ^§^	*p*	*partial* η*^2^*
Somatization	SH	Low	217	18.31 (6.48)	159.75	<0.001	0.31
		High	144	29.88 (10.89)			
		Total	361	22.93 (10.22)			
	SE	Low	229	23.33 (8.96)	0.42	n.s.	0.001
		High	118	22.69 (8.21)			
		Total	347	23.12 (8.71)			
Obsessive-Compulsive	SH	Low	217	18.15 (6.59)	321.16	<0.001	0.47
		High	144	32.16 (8.19)			
		Total	361	23.74 (10.00)			
	SE	Low	229	26.07 (8.51)	28.22	<0.001	0.08
		High	118	21.16 (7.42)			
		Total	347	24.40 (8.47)			
Interpersonal Sensitivity	SH	Low	217	14.30 (5.04)	189.87	<0.001	0.35
		High	144	23.62 (7.81)			
		Total	361	18.02 (7.77)			
	SE	Low	229	19.47 (7.94)	4.47	0.035	0.01
		High	118	17.63 (7.16)			
		Total	347	18.84 (7.72)			
Depression	SH	Low	217	21.87 (7.90)	419.59	<0.001	0.54
		High	144	41.72 (10.48)			
		Total	361	29.79 (13.26)			
	SE	Low	229	32.28 (12.19)	8.59	0.004	0.02
		High	118	28.46 (10.10)			
		Total	347	30.98 (11.65)			
Anxiety	SH	Low	217	14.64 (5.23)	342.63	<0.001	0.49
		High	144	27.91 (8.39)			
		Total	361	19.93 (9.31)			
	SE	Low	229	20.31 (8.15)	0.37	n.s.	0.001
		High	118	19.75 (8.15)			
		Total	347	20.12 (8.14)			
Hostility	SH	Low	217	9.62 (3.64)	113.13	<0.001	0.24
		High	144	14.49 (5.06)			
		Total	361	11.57 (4.88)			
	SE	Low	229	12.48 (5.25)	7.63	0.006	0.02
		High	118	10.96 (3.97)			
		Total	347	11.96 (4.90)			
Phobic Anxiety	SH	Low	217	8.49 (2.54)	76.15	<0.001	0.18
		High	144	12.07 (5.18)			
		Total	361	9.92 (4.19)			
	SE	Low	229	10.18 (4.16)	1.90	n.s.	0.005
		High	118	9.57 (3.48)			
		Total	347	9.97 (3.95)			
Paranoid Ideation	SH	Low	217	10.41 (3.97)	94.49	<0.001	0.21
		High	144	15.18 (5.33)			
		Total	361	12.32 (5.12)			
	SE	Low	229	13.02 (5.34)	1.94	n.s.	0.006
		High	118	12.20 (4.86)			
		Total	347	12.74 (5.19)			
Psychoticism	SH	Low	217	13.96 (4.80)	112.82	<0.001	0.24
		High	144	20.76 (7.37)			
		Total	361	16.68 (6.82)			
	SE	Low	229	18.03 (6.93)	13.99	<0.001	0.04
	High	118	15.29 (5.44)			
	Total	347	17.10 (6.58)			

*Psychopathology scales by low and high Studyholism (SH) and Study Engagement (SE). ^§^ = for Studyholism, df = 1,359; for Study Engagement, df = 1,345.*

Next, we performed two MANOVAs on the subsample of participants who also filled the SSS-V (*n* = 717) to analyze if there are differences in sensation seeking between students characterized by high (*n* = 115) and low (*n* = 100) levels of Studyholism, as well as by high (*n* = 81) and low (*n* = 110) levels of Study Engagement.

The multivariate tests highlighted a statistically significant effect on sensation seeking for both Studyholism [*F*(4,210) = 3.18, *p* = 0.015, η^2^ = 0.06] and Study Engagement [*F*(4,186) = 12.48, *p* < 0.001, η^2^ = 0.21]. Moreover, follow-up ANOVAs highlighted statistically significant differences in all the SSS-V scales for both the SI-10 scales: students demonstrating high levels of Studyholism and Study Engagement have lower scores on all the sensation seeking scales. Table 3 shows the results of follow-up ANOVAs analyses.

**TABLE 3:** Follow-up ANOVAs.

SSS-V		Level	*n*	*M (SD)*	*F* ^§^	*p*	*partial* η*^2^*
Boredom Susceptibility	SH	Low	100	3.60 (1.84)	5.51	0.020	0.03
		High	115	3.03 (1.74)			
		Total	215	3.29 (1.81)			
	SE	Low	110	3.75 (1.72)	17.81	<0.001	0.09
		High	81	2.69 (1.72)			
		Total	191	3.30 (1.80)			
Disinhibition	SH	Low	100	5.00 (2.47)	7.58	0.006	0.03
		High	115	4.13 (2.16)			
		Total	215	4.53 (2.35)			
	SE	Low	110	5.53 (2.23)	41.69	<0.001	0.18
		High	81	3.44 (2.17)			
		Total	191	4.64 (2.43)			
Experience Seeking	SH	Low	100	6.49 (1.95)	7.35	0.007	0.03
		High	115	5.77 (1.92)			
		Total	215	6.11 (1.96)			
	SE	Low	110	6.64 (1.93)	20.71	<0.001	0.10
		High	81	5.37 (1.85)			
		Total	191	6.10 (2.00)			
Thrill and Adventure Seeking	SH	Low	100	6.01 (2.75)	4.23	0.041	0.02
		High	115	5.22 (2.88)			
		Total	215	5.59 (2.84)			
	SE	Low	110	6.22 (2.86)	7.95	0.005	0.04
		High	81	5.07 (2.65)			
		Total	191	5.73 (2.82)			

*Sensation seeking scales by low and high Studyholism (SH) and Study Engagement (SE). ^§^ = for Studyholism, df = 1,213; for Study Engagement, df = 1,189.*

### Disengaged and Engaged Studyholics: Differences in Psychopathology and Sensation Seeking

In the total sample, the percentage of the four types of student suggested by Loscalzo and Giannini (2017b,2020c) are as follows: Disengaged Student, *n* = 48, 3.9%; Disengaged Studyholic, *n* = 18, 1.5%; Engaged Student, *n* = 18, 1.5%; Engaged Studyholic, *n* = 21, 1.7%. Hence, to compare students showing Engaged and Disengaged Studyholism, we must use non-parametric analyses. In fact, for sensation seeking, the number of students belonging to the Engaged and Disengaged types were even lower (15 and 13 participants, respectively). Therefore, we performed Mann–Whitney tests (with Yates’ continuity correction).

Regarding psychopathology, the results highlighted a statistically significant difference between the two types of Studyholic on the obsessive-compulsive scale only: χ*^2^*(1) = 9.24, *p* = 0.002. More specifically, students showing Engaged Studyholism have lower levels of these symptoms (Median = 25.00) than those demonstrating Disengaged Studyholism (Median = 33.50). For sensation seeking, the Mann–Whitney tests showed no statistically significant differences between the two types of Studyholics.

## Discussion

The present study analyses how two types of HSI—Studyholism (or obsession toward study, namely a new potential OCD-related disorder; Loscalzo and Giannini, 2017b) and Study Engagement—relate with internalizing and externalizing symptoms and sensation seeking.

First, the correlations among the study variables provided preliminary evidence for the positive association between Studyholism and psychopathology, with the obsession component (compared to compulsions and social impairment) having the highest values of correlation. Study Engagement, instead, has (low) negative correlations with just a few of the psychopathology scales. Finally, sensation seeking (a feature of substance addictions, which are externalizing disorders) has low and negative correlation values with both types of HSI.

In line with this, the first path analysis model showed that Studyholism positively predicts all the internalizing and externalizing symptoms, while Study Engagement negatively predicts them (except for somatization and anxiety). Therefore, as expected, Studyholism is associated with worse mental health, while Study Engagement is associated with better mental health; though, the β values are higher for Studyholism, suggesting that *the protective role of Study Engagement is smaller than the risk factor posed by Studyholism.* Also, Study Engagement does not (negatively) predict all the symptoms. Hence, it is vital to address Studyholism aiming to reduce it for preventive purposes, as fostering Study Engagement might not suffice. In fact, high levels of Study Engagement might coexist with high levels of Studyholism. Therefore, to promote better mental health in students, it is critical to reduce the levels of Studyholism. From a theoretical perspective, the results concerning the psychopathology scale whose variance is explained the most by HSI (that is, the obsessive-compulsive scale, followed by two other internalizing scales: depression and anxiety) might support the conceptualization of Studyholism as an OCD-related disorder or, more generally, an internalizing disorder. Though, it should be considered that participants have not been screened for the presence of clinical diagnoses.

Finally, in line with a previous study on Italian youths, which highlighted that Studyholism does not predict a higher GPA (in contrast with Study Engagement; Loscalzo and Giannini, 2019a), Studyholism does not predict the number of exams given (regardless of the positive or negative outcome of the exams), while Study Engagement is a positive predictor of this variable. Thus, *in line with the definition of Studyholism as a potential new clinical disorder (hence, as a condition associated with functional impairment), the time spent obsessing with studying is not associated with higher academic productivity.* Even if youths and adolescents spend much time studying (Loscalzo and Giannini, 2019a; Loscalzo, 2021), they do not have a higher GPA (Loscalzo and Giannini, 2019a; Loscalzo, 2021) and, based on the current study, they might even be unable to take their exams. Consequently, even if it is imperative to avoid over-pathologizing a common behavior, such as studying, we might suggest that overstudying might be conceptualized as a clinical condition, if associated with academic, social, and well-being impairment (and if it is not better explained by the symptoms of other established clinical disorders), as suggested by Loscalzo and Giannini’s (2020a) DSM-like tentative criteria. For example, Loscalzo and Giannini (2020a) underlined that a diagnosis of Studyholism should not be posed if the compulsion to study is a consequence of obsessions not study-related (i.e., OCD disorder) or of the fear of a negative evaluation (i.e., social anxiety disorder).

The path analysis model distinguishing the three Studyholism components (i.e., obsessions, compulsions, and social impairment) confirmed the results concerning the obsessive factor as analyzed through the previous model (that measured Studyholism in its obsessive symptoms only). Moreover, it highlighted the importance of distinguishing the obsessive and compulsive components of problematic overstudying, as they might have different relationships with the same variables. Compulsions, in fact, predicts (negatively) only phobic anxiety, suggesting that *it is the obsessive component to be critical in predicting psychological impairment.* Moreover, compulsions positively predict the number of exams given. These results are in line with the findings by Loscalzo and Giannini (2020a) concerning GPA: compulsions (and social impairment) have a positive correlation with GPA, while obsessions have a (lower) negative correlation with GPA. Therefore, aiming to unveil the internalizing and/or externalizing nature of problematic overstudying, it is vital to distinguish between the two components: compulsions are a common feature of OCD and substance/behavioral addictions, while obsession is the specific feature of OCD. More specifically, as previously pointed out by Loscalzo and Giannini (2020a, p. 10), “the compulsion component could be present both in Studyholics/Study Addicted and Engaged students since the inner compulsion to study corresponds to the high time investment in the study that characterizes both Studyholism and Study Engagement. Hence, it could be the obsession component to be determinant for the arising of Studyholism.” Therefore, *the current study further supports the conceptualization of problematic overstudying as an OCD-related disorder* (or, at least, as an internalizing disorder) since it is the obsessive component (and not the compulsive one) to be critical to predict higher psychopathology and to be associated with worse academic performance (i.e., it is not associated with a higher GPA or number of exams taken).

As additional support to the conceptualization of Studyholism as an OCD-related disorder, the path model analyzing psychopathology and sensation seeking as antecedents of HSI showed that *Studyholism is positively predicted by three internalizing scales (obsessive-compulsive, depression, and anxiety), while two externalizing variables (psychoticism and boredom susceptibility) negatively predict it.* Moreover, the model with the three Studyholism components showed that the predictors explain a good percentage of variance just for obsessions (with the same three internalizing variables playing the greatest role—there are just a few slight differences in the predictors compared to the previous model, mainly concerning the externalizing variables). Interestingly, compulsions (the feature that might be present both in OCD and substance/behavior disorders) is predicted positively by anxiety, and negatively, by two externalizing variables (i.e., hostility and disinhibition). Hence, we suggest that this *provides further evidence for the conceptualization of problematic overstudying as an internalizing disorder (and not as a behavioral addiction) since even the compulsion component is predicted by internalizing features and, negatively, by externalizing variables.*

Compared to the study by Loscalzo and Giannini (2020b), it is interesting to note that there is a critical difference in the predictors of Studyholism and Workaholism, the last one being predicted by an externalizing scale only: psychoticism. Based on their findings, Loscalzo and Giannini (2020b) suggested considering the possibility (among others) of conceptualizing problematic overworking as the declination at work of a personality disorder (such as the schizoid or obsessive-compulsive personality disorder). Hence, *we have found further support for the recommendation of Loscalzo and Giannini (2017b,2019b) to postulate two different theorizations and operationalizations of problematic overworking and overstudying* since, even if both are related to the main activity of workers/students, besides similarities, they also have critical differences. In fact, while Psychoticism positively predicts Workaholism, it negatively predicts Studyholism. Also, Studyholism is predicted by other internalizing (positively) and externalizing (negatively) symptoms.

There are some differences also concerning Study Engagement and Work Engagement. The present study found that Study Engagement is negatively predicted by obsessive-compulsive symptoms, hostility, boredom susceptibility, and disinhibition; though, it is also positively predicted by paranoid ideation and anxiety. Instead, Loscalzo and Giannini (2020b) found that Work Engagement is negatively predicted by depression and boredom susceptibility, and positively, by somatization: hence, they suggested that Work Engagement might be a coping strategy for workers experiencing somatic symptoms. Study Engagement as well might be seen as a coping strategy with distressing symptoms; though, it seems that students use this strategy for managing anxiety and paranoid symptoms (instead of somatic symptoms). Again, these findings suggest that *the construct of Work Engagement might not be transferred into the study area without a specific conceptualization.*

Hence, in analyzing the two types of HSI, we advocate that it is advantageous to use the Studyholism Inventory (SI-10; Loscalzo et al., 2018) and the Studyholism Inventory—Extended version (SI-15; Loscalzo and Giannini, 2020a). In the literature, there are available the students’ version of the following two widely used instruments for the evaluation of Workaholism and Work Engagement: the Bergen Work Addiction Scale (BWAS; Andreassen et al., 2012) and the Utrecht Work Engagement Scale (UWES-9; Schaufeli et al., 2006). These instruments are the Bergen Study Addiction Scale (BSTAS; Atroszko et al., 2015) and the Utrecht Work Engagement Scale, Short and Student version (UWES-S; Schaufeli and Bakker, 2004). Though, compared to these instruments, the SI-10 and SI-15 have been created from a pool of items specifically addressing study behaviors. Therefore, we adopted the SI-10 and the SI-15, even if there were available Italian versions of the BStAS (Loscalzo and Giannini, 2018c) and the UWES-S (Loscalzo and Giannini, 2019c). Also, concerning the BStAS, it is based on the addiction framework instead of the obsessive and HSI framework.

Besides the four path analysis models, we performed multivariate analyses to compare students characterized by high and low levels of Studyholism/Study Engagement on psychopathology and sensation seeking. We found that students with high levels of Studyholism have higher scores on all the psychopathology scales than students with low levels of Studyholism, *providing further support to the psychological impairment associated with high Studyholism.* Moreover, students with high Study Engagement have lower levels of obsessive-compulsive symptoms, interpersonal sensitivity, depression, hostility, and psychoticism. Again, *even if study engagement has a protective role concerning psychopathology, its importance is lower than Studyholism since it influences fewer symptoms.* Hence, it is confirmed that preventive interventions should primarily address Studyholism. About sensation seeking, students with high levels of Studyholism and Study Engagement have lower scores on all the sensation seeking scales compared, respectively, to students with low levels of Studyholism and Study Engagement. Hence, we speculate that this provides further evidence to the conceptualization of Studyholism as an OCD-related disorder (or, at least, as an internalizing disorder), since if it would have been better conceptualized as an externalizing disorder (such as a behavioral addiction), higher levels of sensation seeking should have arisen in students with high Studyholism.

Finally, we analyzed if students demonstrating Engaged and Disengaged Studyholism, that is, the two types of Studyholics (Loscalzo and Giannini, 2017b,2020c), differ on psychopathology and sensation seeking. Interestingly, we found a statistically significant difference only for obsessive-compulsive symptoms: students showing Engaged Studyholism have lower levels of these symptoms than those showing Disengaged Studyholism. This result supports the theorization of Loscalzo and Giannini (2017b) about potential differences between the two types of Studyholics regarding their antecedents and outcomes. In fact, in line with previous studies (Loscalzo and Giannini, 2019a; Loscalzo, 2021), we found not only similarities, but also a critical difference, between the two types. Moreover, since the only difference is on *Obsessive-Compulsive symptoms*, this might provide additional support to the OCD-related conceptualization (in contrast with the behavioral addiction one). These symptoms proved to be *critical for Studyholism both in general and for differentiating between the two types of Studyholic.*

In conclusion, among the limitations of this study, the participants are mainly women who were from Central Italy. Moreover, the scale used for evaluating sensation seeking, even if used by previous Italian studies (e.g., Tonetti et al., 2010; Loscalzo and Giannini, 2020b), has not been validated. Therefore, even though using this scale allowed us to compare the results with Loscalzo and Giannini (2020b), we suggest analyzing further the relationship between HSI and sensation seeking using other scales. Moreover, for assessing psychopathology, we used a self-report scale. Future studies could evaluate psychopathology through structured diagnostic clinical interviews. Also, we did not evaluate participants for the presence of established clinical diagnoses. Finally, it should be considered that the conclusions about the antecedents and outcomes of HSI are based on cross-sectional data. Future studies should employ longitudinal designs to deepen the analysis of the directionality between psychopathology, sensation seeking, and Studyholism.

Despite these limitations, the present study has the main merit of shedding light on a new potential clinical condition that Loscalzo and Giannini (2017b) conceptualized as more similar to an obsession than an addiction (Atroszko et al., 2015). By analyzing the role of Studyholism (and Study Engagement) as an antecedent of psychopathology and the number of exams given (regardless of the outcome), it showed that Studyholism is associated with psychological and academic impairment, hence supporting the conceptualization of problematic overstudying as a clinical condition. For almost all the clinical diagnoses, in fact, an impairment is required in important areas of the individual (American Psychiatric Association [APA], 2013). Also, concerning psychological health, it showed that the risk factor posed by Studyholism is higher than the protective role of Study Engagement. Hence, for counseling services located at universities, it would be important to implement interventions to detect students with high levels of Studyholism and provide individual and group interventions intended to decrease Studyholism levels. Moreover, the analysis of psychopathology and sensation seeking as antecedents of HSI provided additional evidence to the conceptualization of Studyholism as an OCD-related disorder, since Studyholism (as evaluated through the SI-10) is positively predicted by three internalizing symptoms (i.e., obsessive-compulsive, depression, and anxiety) and negatively predicted by two externalizing variables (i.e., psychoticism and boredom susceptibility). Furthermore, even the compulsion component of Studyholism (as evaluated through the SI-15) is predicted by internalizing features, and negatively, by externalizing variables. Next, the comparison of the current results with that of Loscalzo and Giannini (2020b) concerning problematic overworking highlighted that Studyholism/problematic overstudying and Workaholism/problematic overworking (and Work/Study Engagement) should be analyzed as different constructs, that is, with different theorizations and operationalizations, instead of merely adapting the Workaholism/Work Addiction construct to studying. Finally, this study provided further evidence about the need to distinguish between Engaged and Disengaged Studyholics since they might have different relationships with the same antecedents and outcomes, with important implications for clinical tailored interventions.

In sum, in line with the previous studies (Loscalzo and Giannini, 2019a; Loscalzo, 2021), this research provided further evidence to the conceptualization of problematic overstudying as a new potential clinical condition that, based on the meager current literature, seems to be better conceptualized as an OCD-related disorder (and not as an addiction), or more generally, as an internalizing (rather than externalizing) disorder. However, as previously stressed by Loscalzo and Giannini (2018a,2019a), the literature concerning problematic overstudying is too scant to reach any firm conclusion; hence, we prompt other scholars interested in students’ well-being to analyze further this new *potential* clinical disorder, aiming to unveil its internalizing and/or externalizing nature and avoiding a confirmatory approach. Also, future studies could implement and test for efficacy interventions aimed at decreasing Studyholism and increasing Study Engagement.

## Data Availability Statement

The datasets analyzed for this study can be received, upon reasonable request and for research purpose only, by writing to YL.

## Ethics Statement

The studies involving human participants were reviewed and approved by the Research Ethics Commission of the University of Florence. The participants provided their informed consent to participate in this study.

## Author Contributions

YL designed the study, performed the literature search and the data analyses, and drafted the manuscript. MG critically revised the design of the study and the manuscript. Both authors contributed to the article and approved the submitted version.

## Conflict of Interest

The authors declare that the research was conducted in the absence of any commercial or financial relationships that could be construed as a potential conflict of interest.

## Publisher’s Note

All claims expressed in this article are solely those of the authors and do not necessarily represent those of their affiliated organizations, or those of the publisher, the editors and the reviewers. Any product that may be evaluated in this article, or claim that may be made by its manufacturer, is not guaranteed or endorsed by the publisher.

## References

[B1] American Psychiatric Association [APA] (2013). *Diagnostic and Statistical Manual of Mental Disorders, (DSM-5).* 5th Edn. Washington, DC: American Psychiatric Association. 10.1176/appi.books.9780890425596

[B2] Andreassen C. S. Griffiths M. D. Hetland J. Pallesen S. (2012). Development of a work addiction scale. *Scand. J. Psychol.* 53 265–272. 10.1111/j.1467-9450.2012.00947.x 22490005

[B3] Andreassen C. S. Pallesen S. Trosheim T. (2018). Workaholism as a mediator between work-related stressors and health outcomes. *Int. J. Environ. Res. Public Health* 15:e73. 10.3390/ijerph15010073 29303969 PMC5800172

[B4] Andreassen C. S. Ursin H. Eriksen H. R. (2007). The relationship between strong motivation to work, “workaholism”, and health. *Psychol. Health* 22 615–629. 10.1080/14768320600941814

[B5] Atroszko P. A. Andreassen C. S. Griffiths M. D. Pallesen S. (2015). Study addiction – a new area of psychological study: conceptualization, assessment, and preliminary empirical findings. *J. Behav. Addict.* 4 75–84. 10.1556/2006.4.2015.007 26014668 PMC4500887

[B6] Auerbach R. P. Alonso J. Axinn W. G. Cuijpers P. Ebert D. D. Green J. G. (2016). Mental disorders among college students in the World Health Organization world mental health surveys. *Psychol. Med.* 46 2955–2970. 10.1017/S0033291716001665 27484622 PMC5129654

[B7] Bartcazk M. Ogińska-Bulik N. (2012). Workaholism and mental health among Polish academic workers. *Int. J. Occup. Saf. Ergon.* 18 3–13. 10.1080/10803548.2012.11076910 22429525

[B8] Billieux J. Schimmenti A. Khazaal Y. Maurage P. Heernen A. (2015). Are we overpathologizing everyday life? A tenable blueprint for behavioral addiction research. *J. Behav. Addict.* 4 119–123. 10.1556/2006.4.2015.009 26014667 PMC4627665

[B9] Blanco C. Okuda M. Wright C. Hasin D. S. Grant B. F. Liu S. (2008). Mental health of college students and their non college-attending peers: results from the national epidemiologic study on alcohol and related conditions. *Arch. Gen. Psychiatry* 65 1429–1437. 10.1001/archpsyc.65.12.1429 19047530 PMC2734947

[B10] Byrne B. M. (2001). *Structural Equation Modeling With AMOS: Basic Concepts, Applications, and programming.* Mahwah, NJ: Lawrence Erlbaum Associates.

[B11] Clark M. A. Michel J. S. Zhdanova L. Pui S. Y. Baltes B. B. (2016). All work and no play? A meta-analytic examination of the correlates and outcomes of workaholism. *J. Manag.* 42 1836–1873. 10.1177/0149206314522301

[B12] Comer J. S. Kendall P. C. Franklin M. E. Hudson J. L. Pimentel S. S. (2004). Obsessing/worrying about the overlap between obsessive-compulsive disorder and generalized anxiety disorder in youth. *Clin. Psychol. Rev.* 24 663–683. 10.1016/j.cpr.2004.04.004 15385093

[B13] Derogatis L. R. (1994). *Symptom Checklist-90-R, SCL-90-R: Administration, Scoring and Procedures Manual.* Minneapolis, MN: National Computer Systems.

[B14] Drum D. J. Brownson C. Burton Denmark A. Smith S. (2009). New data on the nature of suicidal crises in college students: shifting the paradigm. *Prof. Psychol. Res. Pract.* 40 213–222. 10.1037/a0014465

[B15] Eisenberg D. Hunt J. Speer N. (2013). Mental health in American colleges and universities: variation across student subgroups and across campuses. *J. Nerv. Ment. Dis.* 201 60–67. 10.1097/NMD.0b013e31827ab077 23274298

[B16] Gallagher R. (2008). *National Survey of Counseling Center Directors. Monograph Series Number 8Q.* Alexandria, VA: The International Association of Counseling Services Inc.

[B17] Haar J. Roche M. (2013). Three-way interaction effects of workaholism on employee well-being: evidence from blue-collar workers in New Zealand. *J. Manag. Organ.* 19 134–149. 10.1017/jmo.2013.10

[B18] Hu L. Bentler P. M. (1999). Cutoff criteria for fit indexes in covariance structure analysis: conventional criteria versus new alternatives. *Struct. Equ. Model.* 6 1–55. 10.1080/10705519909540118

[B19] Ibrahim A. K. Kelly S. J. Adams C. E. Glazebrook C. (2013). A systematic review of studies of depression prevalence in university students. *J. Psychiatry Res.* 47 391–400. 10.1016/j.jpsychires.2012.11.015 23260171

[B20] Kardefelt-Winther D. (2015). Commentary on: are we overpathologizing everyday life? A tenable blueprint for behavioral addiction research. Problems with atheoretical and confirmatory research approaches in the study of behavioral addictions. *J. Behav. Addict.* 4 126–129. 10.1556/2006.4.2015.019 26551896 PMC4627667

[B21] Keyes C. L. Eisenberg D. Perry G. S. Dube S. R. Kroenke K. Dhingra S. S. (2012). The relationship of level of positive mental health with current mental disorders in predicting suicidal behavior and academic impairment in college students. *J. Am. Coll. Health* 60 126–133. 10.1080/07448481.2011.608393 22316409

[B22] Lawendowski R. Bereznowksi P. Wróbel W. Kierzkowski M. Atroszko P. A. (2020). Study addiction among musicians: measurement, and relationship with personality, social anxiety, performance, and psychosocial functioning. *Music. Sci.* 24 449–474. 10.1177/1029864918822138

[B23] Linden-Carmichael A. N. Stamates A. L. Sheehan B. E. Lau-Barraco C. (2016). Molly users versus non users in a sample of college alcohol drinkers: differences in substance-related harms and sensation seeking. *Subst. Abus.* 37 474–479. 10.1080/08897077.2015.1137536 26820396 PMC4993673

[B24] Loscalzo Y. (2019). Heavy study investment in college students: studyholism and study engagement prevalence. *Appl. Psychol. Bull.* 286 55–61. 10.26387/bpa.286.4

[B25] Loscalzo Y. (2021). Studyholism and study engagement: what about the role of perfectionism, worry, overstudy climate, and type of school in adolescence? *Int. J. Environ. Res. Public Health* 18:910. 10.3390/ijerph18030910 33494372 PMC7908290

[B26] Loscalzo Y. Giani J. Giannini M. (2021). Heavy Study Investment in Pre-Adolescence and Adolescence: Psychometric properties of the Studyholism Inventory (SI-10). Manuscript submitted for publication.

[B27] Loscalzo Y. Giannini M. (2017a). Clinical conceptualization of workaholism: a comprehensive model. *Organ. Psychol. Rev.* 7 306–329. 10.1177/2041386617734299

[B28] Loscalzo Y. Giannini M. (2017b). “Studyholism or study addiction? A comprehensive model for a possible new clinical condition,” in *Advances in Psychological Research*, ed. Columbus A. M. (New York, NY: Nova Publisher), 19–37.

[B29] Loscalzo Y. Giannini M. (2018a). Problematic overstudying: studyholism or study addiction? Commentary on: ten myths about work addiction. *J. Behav. Addict.* 7 867–870. 10.1556/2006.7.2018.124 30541335 PMC6376362

[B30] Loscalzo Y. Giannini M. (2018b). Response to: theoretical and methodological issues in the research on study addiction with relevance to the debate on conceptualising behavioural addictions: Atroszko (2018). *Psychiatr. Psychol. Klin.* 18 426–430. 10.15557/PiPK.2018.0051

[B31] Loscalzo Y. Giannini M. (2018c). The bergen study addiction scale: psychometric properties of the italian version. a pilot study. *Psychiatr. Psychol. Klin.* 18 271–275. 10.15557/PiPK.2018.0033

[B32] Loscalzo Y. Giannini M. (2019a). Heavy study investment in italian college student. An analysis of Loscalzo and Giannini’s (2017) studyholism comprehensive model. *Front. Psychiatry* 10:489. 10.3389/fpsyt.2019.00489 PMC6651580 31379617

[B33] Loscalzo Y. Giannini M. (2019b). What type of worker are you? Work-Related Inventory (WI-10): a comprehensive instrument for the measurement of workaholism. *Work* 62 383–392. 30856144 10.3233/WOR-192875

[B34] Loscalzo Y. Giannini M. (2019c). Study engagement in Italian University students: a confirmatory factor analysis of the Utrecht Work Engagement Scale – Student version. *Soc. Indic. Res.* 142 845–854. 10.1007/s11205-018-1943-y

[B35] Loscalzo Y. Giannini M. (2020a). When studying becomes an obsession: the studyholism inventory-extended version (SI-15). *Curr. Psychol.* 10.1007/s12144-020-01168-3

[B36] Loscalzo Y. Giannini M. (2020b). Heavy work investment and psychopathology: internalizing and externalizing disorders as antecedents and outcomes. *Amfiteatru Econ.* 22 1301–1324. 10.24818/EA/2020/S14/1301

[B37] Loscalzo Y. Giannini M. (2020c). Studyholism Inventory (SI-10): a short instrument for evaluating study obsession in the heavy study investment framework. *Eur. J. Psychol.* 16 688–706. 33680206 10.5964/ejop.v16i4.1911 PMC7909487

[B38] Loscalzo Y. Giannini M. Golonka K. (2018). “Studyholism Inventory (SI-10): psychometric properties of the Italian and Polish versions,” in *Resilience and Health. Challenges for an Individual, Family and Community*, Vol. 2018 eds Ostrowski T. Piasecka B. Gerc K. (Krakow: Jagiellonian University Press), 205–217.

[B39] Megivern D. Pellerito C. Mowbray C. (2003). Barriers to higher education for individuals with psychiatric disabilities. *Psychiatr. Rehabilit. J.* 26 217–232. 10.2975/26.2003.217.231 12653444

[B40] Meil W. M. LaPorte D. J. Milles J. A. Sesti A. N. Collings S. M. Stiver A. G. (2016). Sensation seeking and executive deficits in relation to alcohol, tobacco, and marijuana use frequency among university students: value of ecologically based measures. *Addicti. Behav.* 62 135–144. 10.1016/j.addbeh.2016.06.014 27355485

[B41] Nie Y. Sun H. (2016). Why do workaholics experience depression? A study with Chinese University teachers. *J. Health Psychol.* 21 2339–2346. 10.1177/1359105315576350 25836332

[B42] Oates W. (1971). *Confession of a Workaholic.* New York, NY: Abingdon.

[B43] Reeve B. B. Hays R. D. Bjorner J. B. Cook K. F. Crane P. K. Teresi J. A. (2007). Psychometric evaluation and calibration of health-related quality of life item banks: plans for the Patient-Reported Outcomes Measurement Information System (PROMIS). *Med. Care* 45 (Suppl. 1), S22–S31. 10.1097/01.mlr.0000250483.85507.04 17443115

[B44] Regehr C. Glancy D. Pitts A. (2013). Interventions to reduce stress in university students: a review and meta-analysis. *J. Affect. Disord.* 148 1–11. 10.1016/j.jad.2012.11.026 23246209

[B45] Rogers A. A. Elam K. K. Chassin L. Sternber A. Bui L. (2018). Proximal and distal effects of sensation seeking and parenting environments on alcohol use trajectories from early adolescence to early adulthood. *J. Youth Adolesc.* 47 2206–2219. 10.1007/s10964-018-0874-x 29905884 PMC6151145

[B46] Rosenthal B. Wilson C. (2008). Mental health services: use and disparity among diverse college students. *J. Am. Coll. Health* 57 61–67. 10.3200/JACH.57.1.61-68 18682347

[B47] Salzer M. S. (2012). A comparative study of campus experiences of college students with mental illnesses versus a general college sample. *J. Am. Coll. Health* 60 1–7. 10.1080/07448481.2011.552537 22171723

[B48] Schaufeli W. B. Bakker A. B. (2004). *Test Manual for the Utrecht Work Engagement Scale.* Unpublished manuscript. The Netherlands: Utrecht University.

[B49] Schaufeli W. B. Bakker A. B. Salanova M. (2006). The measurement of work engagement with a short questionnaire. *Educ. Psychol. Meas.* 66 701–716. 10.1177/0013164405282471

[B50] Shimazu A. Schaufeli W. B. Kubota K. Watanabe K. Kawakami N. (2018). Is too much work engagement detrimental? Linear or curvilinear effects on mental health and job performance. *PLoS One* 13:e0208684. 10.1371/journal.pone.0208684 30586369 PMC6306155

[B51] Snir R. Harpaz I. (2012). Beyond workaholism: towards a general model of heavy work investment. *Hum. Resourc. Manag. Rev.* 22 232–243. 10.1016/j.hrmr.2011.11.011

[B52] Storrie K. Ahern K. Tuckett A. (2010). A systematic review: students with mental health problems - a growing problem. *Int. J. Nurs. Pract.* 16 1–6. 10.1111/j.1440-172X.2009.01813.x 20158541

[B53] Strepparava M. G. Iacchia E. (2012). *Psicopatologia Cognitiva dello Sviluppo. Bambini Difficili o Relazioni Difficili? [Cognitive Developmental Psychopathology. Difficult Children or Difficult Relationships ?].* Milano: Raffaello Cortina Editore.

[B54] Sussman S. (2012). Workaholism: a review. *Addict. Res. Theory* S6, 1–18. 10.4172/2155-6105.S6-001 24273685 PMC3835604

[B55] Tonetti L. Adan A. Caci H. De Pascalis V. Fabbri M. Natale V. (2010). Morningness-eveningness preference and sensation seeking. *Eur. Psychiatry* 25 111–115. 10.1016/j.eurpsy.2009.09.007 19926258

[B56] Zuckerman M. (1994). *Behavioral Expressions and Biosocial Bases of Sensation Seeking.* New York, NY: Cambridge University Press.

[B57] Zuckerman M. Eysenck S. B. G. Eysenck H. J. (1978). Sensation seeking in England and America: cross-cultural, age, and sex comparisons. *J. Consult. Clin. Psychol.* 46 139–149. 10.1037/0022-006X.46.1.139 627648

